# The skin prick test – European standards

**DOI:** 10.1186/2045-7022-3-3

**Published:** 2013-02-01

**Authors:** Lucie Heinzerling, Adriano Mari, Karl-Christian Bergmann, Megon Bresciani, Guido Burbach, Ulf Darsow, Stephen Durham, Wytske Fokkens, Mark Gjomarkaj, Tari Haahtela, Ana Todo Bom, Stefan Wöhrl, Howard Maibach, Richard Lockey

**Affiliations:** 1 Department of Dermatology, University Hospital Erlangen, 91054 Erlangen, Germany; 2 Board Member of the EAACI Allergy Diagnosis Interest Group, IDI-IRCCS, Center for Molecular Allergology, Rome, Italy; 3 Department of Dermatology and Allergy, Charité Universitätsmedizin-Berlin, Berlin, Germany; 4 Consiglio Nazionale delle Ricerche, Rome, Italy; 5 Department of Dermatology and Allergy Biederstein and Division of Environmental Dermatology and Center of Allergy and Environment (ZAUM), Technical University, Munich, Germany; 6 Department of Respiratory Medicine, Royal Brompton Hospital, London, UK; 7 Department of Otorhinolaryngology, Academic Medical Centre, Amsterdam, Netherlands; 8 Consiglio Nazionale delle Ricerche, Palermo, Italy; 9 Skin and Allergy Hospital, University Central Hospital, Helsinki, Finland; 10 ImunoAlergologia, Coimbra University, Coimbra, Portugal; 11 Department of Dermatology, Medical University of Vienna, Vienna, Austria & Floridsdorf Allergy Centre (FAZ), Vienna, Austria; 12 Department of Dermatology, University of California, San Francisco, California, USA; 13 Division of Allergy & Immunology, University of South Florida College of Medicine, Tampa, Florida, USA

**Keywords:** Sensitization, Inhalant allergens, Skin prick test panel, Aallergies, Type I allergy, Diagnostic test, Asthma

## Abstract

Skin prick testing is an essential test procedure to confirm sensitization in IgE-mediated allergic disease in subjects with rhinoconjunctivitis, asthma, urticaria, anapylaxis, atopic eczema and food and drug allergy. This manuscript reviews the available evidence including Medline and Embase searches, abstracts of international allergy meetings and position papers from the world allergy literature. The recommended method of prick testing includes the appropriate use of specific allergen extracts, positive and negative controls, interpretation of the tests after 15 – 20 minutes of application, with a positive result defined as a wheal ≥3 mm diameter. A standard prick test panel for Europe for inhalants is proposed and includes hazel (*Corylus avellana*), alder (*Alnus incana*), birch (*Betula alba*), plane (*Platanus vulgaris*), cypress (*Cupressus sempervirens*), grass mix (*Poa pratensis*, *Dactilis glomerata*, *Lolium perenne*, *Phleum pratense*, *Festuca pratensis*, *Helictotrichon pretense*), Olive (*Olea europaea*), mugwort (*Artemisia vulgaris*), ragweed (*Ambrosia artemisiifolia*), *Alternaria alternata* (*tenuis*), *Cladosporium herbarum*, *Aspergillus fumigatus*, *Parietaria*, cat, dog, *Dermatophagoides pteronyssinus*, *Dermatophagoides farinae*, and cockroach (*Blatella germanica*). Standardization of the skin test procedures and standard panels for different geographic locations are encouraged worldwide to permit better comparisons for diagnostic, clinical and research purposes.

## 

Skin prick testing (SPT) is a reliable method to diagnose IgE-mediated allergic disease in patients with rhinoconjunctivitis, asthma, urticaria, anapylaxis, atopic eczema and suspected food and drug allergy. It provides evidence for sensitization and can help to confirm the diagnosis of a suspected type I allergy. It is minimally invasive, inexpensive, results are immediately available and when carried out by trained health professionals, reproducible. Since the first publication about SPT by Helmtraud Ebruster in 1959 [1], who extensively researched this diagnostic test, it has been used as a primary diagnostic tool to detect type I hypersensitivity reactions. Although the principle of SPT still largely resembles the original methods described, a wide array of interpretations and modifications has led to diminished comparability when SPT results are reported. In addition, the different kind of extracts used in various countries makes comparison of data difficult.

The Global Allergy and Asthma European Network (GA^2^LEN) is a network of research investigators formed to augment the cooperation of allergy and asthma research throughout Europe. The purpose of the GA^2^LEN quality management is to standardize procedures used to diagnose and treat allergic diseases. A survey was conducted to assess SPT practices at the different participating centres at the debut of the program. Although there were similarities in technique for SPT, e.g., the use of positive and negative controls and requesting information from the patient about medications that could interfere with test results, there were also striking differences [2]. These investigators realized that standardization of SPT procedures is desirable so that findings from clinical practice and research become more comparable. Therefore, a GA^2^LEN protocol was developed using a common panel of inhalant allergens (Table 1) and a standard operating procedure to perform and appropriately interpret SPT results based on published practice guidelines, the European Academy of Allergy and Clinical Immunology (EAACI) Position Paper, the Nordic standards and the International Study of Asthma and Allergies in Childhood (ISAAC) phase II protocol. The large multicenter GA^2^LEN study was carried out in 17 centers in 14 countries and showed that a proposed standard protocol and allergen panel for SPT are feasible [3,4].

**Table 1:** Standard prick test panel for inhalant allergens

**Allergen**/**control**	
Histamindihydrochloride 0,1 % (positive control)	
NaCl 0.9% (negative control)	
**Hazel**	*Corylus avellana*
**Alder**	*Alnus incana*
**Birch**	*Betula alba*
**Plane**	*Platanus vulgaris*
**Cypress**	*Cupressus sempervirens*
**Grass mix**	smooth meadow grass/*Poa pratensis*, cock’s foot grass/*Dactilis glomerata*, perennial rye grass/*Lolium perenne*, timothy grass/*Phleum pratense*, meadow fescue/*Festuca pratensis*, meadow oat grass/*Helictotrichon pretense*
**Olive**	*Olea europaea*
**Mugwort**	*Artemisia vulgaris*
**Ragweed**	*Ambrosia artemisiifolia*
***Alternaria***	*Alternaria alternata* (*tenuis*)
***Cladosporium***	*Cladosporium herbarum*
***Aspergillus***	*Aspergillus fumigatus*
***Parietaria***	*Parietaria*
**Cat**	
**Dog**	
***Dermatophagoides pteronyssinus***	
***Dermatophagoides farinae***	
***Blatella***	*Blatella germanica*

## Indication for SPT

SPT is indicated if a type I (immediate type) allergy is suspected, based on the medical history and clinical symptoms; they can identify sensitivity to inhalant, food, drug or occupational allergens. SPTs thus provide objective confirmation of sensitivity, whereas the relevance of such sensitivity to allergens should always be carefully interpreted in the light of the clinical history so that appropriate advice concerning avoidance measures can be given and, as necessary, the correct allergen(s) prescribed for specific immunotherapy (SIT). SPT results correlate with those of nasal challenge which may also be used as a surrogate to test clinically relevant sensitization [5].

Another indication of SPT is to screen for a predisposition to develop atopic diseases, which can be done with a limited number of allergens, or to identify all sensitized subjects in a given population. SPT also can be used in epidemiologic studies to determine trends in sensitization rates or regional differences and to help standardize allergen extracts.

SPT is used to test adults and children from birth onwards. Repeated testing may be necessary in order to detect new sensitizations, especially in children, when symptoms change, or if new environmental allergens are suspected.

## General principle in SPT

SPT interpretation utilizes the presence and degree of cutaneous reactivity as a surrogate marker for sensitization within target organs, i.e., eyes, nose, lung, gut and skin. When relevant allergens are introduced into the skin, specific IgE bound to the surface receptors on mast cells are cross-linked, mast cells degranulate, and histamine and other mediators are released. This produces a wheal and flare response which can be quantitated. Many different allergens can be tested simultaneously because the resultant reaction to a specific allergen is localized to the immediate area of the SPT.

## Comparison with other methods

The chief advantage of SPT as compared to an *in vitro* measurement of specific IgE antibodies is that the test can be interpreted within 15 to 20 minutes after the reagent is applied to the skin. Moreover, the test gives a visual indication of the sensitivity which can be used in order to impact the patient’s behavior. SPT can also be utilized to test less common allergens, such as certain medications, and fresh fruits and vegetables where no specific IgE antibody measurements are available.

The skin scratch test, first described by Blackley in 1873 [6], is not recommended as a test modality for inhalant or food allergens since results are more difficult to interpret and standardize. Scratch testing may result in varying quantities of allergen absorbed, mechanical irritation of the skin [7] , bleeding at the test site, and carries a higher risk of inducing a systemic allergic reaction.

The *in vitro* measurement of specific IgE antibodies [8-10] is an important complementary tool to diagnose type I allergy, especially in subjects who cannot undergo SPT. For example, SPT is not practical in patients who have extensive eczema, dermographism, urticaria, or who are taking antihistamines or other medications which interfere with the proper interpretation of the test results (Table 2). *In vitro* test methods may be less sensitive [11,12] and/or less specific [13,14] than SPT depending on the method utilized and the allergens employed. Furthermore, in subjects with very high total serum IgE antibodies, low levels of specific IgE antibodies of doubtful clinical relevance are often detected. Concordance between *in vitro* specific IgE antibody assays and SPT results is between 85% and 95%, depending on the allergen being tested [15-18] and the method used to detect specific IgE [19-21]. In a study of over 8000 subjects, SPT versus quantitation of specific IgE antibodies, for example, with the CAP FEIA technology (Phadiatop®, Pharmacia, Uppsala, Sweden), had the best positive predictive value to determine clinical allergy for respiratory allergic diseases [22]. Moreover, SPT provides immediate information versus *in vitro* test results which may not be available for days or weeks. Thus, SPT has greater flexibility and is usually less costly.

**Table 2:** **Potential interference of medications with the skin test reaction** (**adapted from Demoly** (**2003**) [23]; **Rueff** (**2010**) [24]**and Position Paper**: **Allergen standardization and skin tests**: **The European Academy of Allergy** (**1993**))

**Drug**	**Suppression**	**Abstinence before testing**	**Reference**
	**0: no evidence; (+): possible, +: slight; ++: medium, +++: strong**		
**Antihistamines**			
1st generation H1-blocker	+++	> 2 days	Dreborg (1989) [25]
Hydroxyzine			
2nd generation H1-blocker	+++	7 days	Devillier (2008) [26]
Cetirizine, Loratadine, etc.			
Ketotifen	+++	> 5 days	
H2-blocker	0 - +	Ø	
			
**Glucocorticosteroids**			
Topical (in test area)	+	> 1 week ^1^	Hammarlund (1990) [27], Pipkorn (1989) [28], Gradman (2008) [29]
Nasal	0	Ø	
Inhaled	0	Ø	
Systemic/short term (up to 10 days)	0 / (+)		
< 50 mg/d Prednisolone-equivalent	0 / (+)	> 3 days	Hammarlund (1990) [30]
> 50 mg/d Prednisolone-equivalent	(+)	> 1 week ^2^	Des Roches (1996) [31]
Systemic/long term (more than 10 days)			
<10 mg/d Prednisolone-equivalent	0	Ø	Olson (1990) [32]
>10 mg/d Prednisolone-equivalent	0	> 3 weeks ^2^	Des Roches (1996) [31]
Topical calcineurin inhibitors	+	> 1 week	Gradman (2008) [29]
**Other systemic drugs**			
Omalizumab	++	> 4 weeks	Noga (2003) [33]
Leukotriene receptor antagonist	0	Ø	Cuhadaroglu (2001) [34], Hill (2003) [35]
Cyclosporin A	0	Ø	Munro (1991) [36]
Theophylline	0	Ø	Spector (1979) [37]
Antidepressants			
Doxepin	++	7 days	Rao (1988) [38]
Desipramine	++	3 days	Rao (1988) [38]
SSRI: Citalopram, Fluoxetin, Sertralin	0	Ø	Isik (2011) [39]
β-adrenergic agonists	0	Ø	Abramowitz (1980) [40], Spector (1979) [39]
Salbutamol, Salmeterol, Bambuterol, Terbutalin	0		Petersen (2003) [41]

^1^ Depends on dosage and length of treatment (> 3 weeks).

^2^ A retrospective study showed no influence of the skin reaction by 10–60 mg prednisone for 2 or more years.

Intradermal skin tests are more sensitive but less specific than SPT [42]. They are more labor-intensive and require more precise techniques. These tests have occasionally been associated with serious systemic allergic reactions and even death from anaphylaxis [43,44]. In clinical practice, SPT tests should always be performed first since a positive test circumvents the necessity for intradermal skin testing. Extracts utilized for intradermal skin testing are less concentrated (1:10–1:1000; 0.00001 μg/ml up to 1 μg/ml [42,45]) than those utilized for SPT and should be free of glycerine, in order to avoid false-positive reactions. In the diagnosis of pollen allergy, several studies indicate that positive intradermal skin tests do not necessarily correlate with clinical symptoms [22,42] whereas there is a very good correlation between SPT results and clinical allergy symptoms [25]. Thus, for the most part, SPT is preferable to intradermal testing, the latter being primarily used for Hymenoptera venom sensitivity, sensitization to medications, and where an allergen is considered historically relevant and in the circumstance that the SPT is negative [46]. Titration tests for Hymenoptera venoms are usually begun at concentrations of 1:1000 or after prior negative SPT using 1:100.

## Performance of the skin prick test

### Preparation, precautions and contraindications

SPT is safe with no reported fatalities in a 5-year USA study [47]. Because systemic allergic reactions and rare deaths have occurred associated with SPT [44,48], a physician or other health care professional and emergency equipment should be immediately available when such tests are performed. This is especially true when testing for a food or medication associated with the onset of anaphylaxis [49]. Systemic side effects are very unlikely for commercially available respiratory allergens [50]. Symptomatic asthma may be a risk factor for exacerbation of asthma associated with testing [44]. When reactions occur, they usually do so within 30 minutes of testing [50]. Measurement of specific IgE antibodies or titrated SPT are sometimes desirable for patients with severe anaphylaxis suspected from a specific allergen to which they are being tested, i.e., peanuts, tree nuts and shellfish. SPT should be performed with extra caution during the respective allergy season when the patient has allergic symptoms, or when baseline tryptase levels are elevated. The latter is a risk factor for anaphylaxis [51]. Likewise, patients, especially those taking a beta blocker, or less often, angiotensin converting enzyme (ACE)-inhibitor, may be at a higher risk because of less response to epinephrine that might be needed to treat a systemic allergic reaction. Relative contraindications for SPT include pregnancy, in view of a remote possibility of inducing a systemic allergic reaction that could induce uterine contractions or necessitate the use of epinephrine (thought to cause constriction of the umbilical artery [52]). SPTs are difficult to perform in patients with severe eczema, dermographism, or who are taking antihistamines or other medications such as certain antidepressants or calcineurin inhibitors (see Table 2) which can interfere with the proper interpretation of the test results. In vitro testing is recommended in these cases. The degree of skin test reactivity can be decreased in subjects with chronic illnesses such as renal failure, or cancer. Furthermore, chronic or acute UV-B radiation of the skin in the test area may reduce the wheal size from SPT [53].

The stability and expiration date of the allergen extracts utilized should always be checked. Test extracts should be stored at +2°C - +8°C when not utilized to maintain stability. Histamine dihydrochloride (10 mg/ml or 0.1%) can be used as a positive control and diluent, as used in the test extracts, as a negative control. For oral allergy syndrome induced by certain foods, raw foods, i.e., fresh fruits and vegetables are preferably used. The skin of the fruit or vegetable is pricked and then the skin of the allergic patient, in order to determine skin test reactivity.

### SPT procedure

Patients should be appropriately screened for asthma, and, where possible, discontinued on medications that interfere with test results, accentuate systemic allergic reactions or render patients less responsive to treatment with epinephrine. In patients with a history of severe systemic allergic reactions to food or drugs, an intravenous line for immediate circulatory access can be recommended. A peak flow of less than 70% in patients with asthma is a relative contraindication. Asthma should be controlled or testing deferred until control is achieved. When testing patients with a history of a severe systemic allergic reactions, skin test titration, first utilizing diluted extracts, is recommended. SPT should ideally be performed at least 4–6 weeks following a systemic allergic reaction, in particular, for Hymenoptera hypersensitivity, since test reactivity may be falsely negative for weeks following such a reaction [24,54].

**Figure 1:**
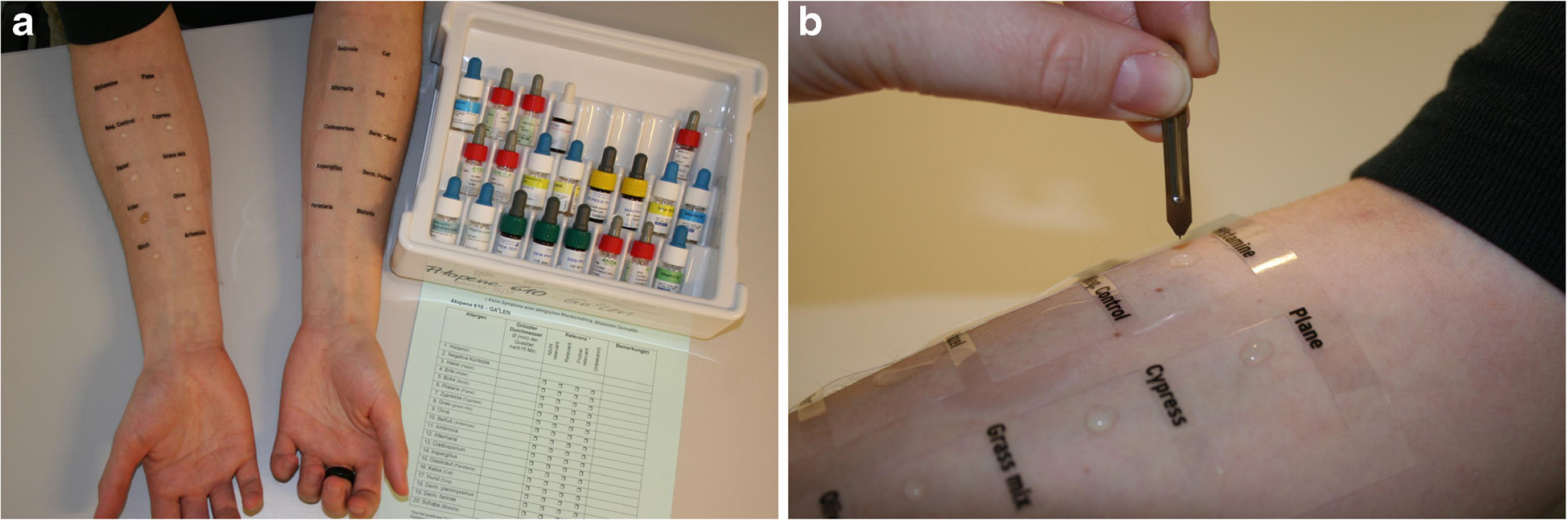
**SPT procedures.** (**a**) Preparation for skin prick test on forearm. (**b**) Prick testing with lancet through a drop of allergen extract.

The location of each allergen can be marked with a pen or by using a test grid on the forearm to properly identify test results (Figure 1a). Tests should be applied to the volar aspect of the forearm, at least 2 – 3 cm from the wrist and the antecubital fossae [52]. The back can also be used for SPT, especially in infants. The skin on the back is more sensitive than the forearm which may result in larger wheals and thus possibly a greater number of positive test results [55]. The distance between two skin prick tests (≥ 2 cm) is critical to avoid false-positive reactions due to direct contamination of a nearby test or secondary to an axon reflex [55]. A drop of each test solution should be placed on the skin in identical order for each subject tested and immediately pricked.

A single-head metal lancet exhibits excellent reproducibility with few false-negative results and is thus the preferred testing instrument for SPT [56-58]. It is pressed through the drop of allergen extract and held against the skin for at least 1 second (Figure 1b), with equal pressure applied for each test. The epithelial layer of the skin should be penetrated without inducing bleeding, which can lead to false-positive results. A new lancet should be utilized for each allergen since wiping a previously used one between tests could result in cross-contamination from the previous allergen tested [59]. Wiping lancets also represents a potential risk factor for the healthcare professional performing the test. Excess solution from drops on the skin can be blotted using a clean tissue. It is important to assure that there is no cross-contamination between drops of different allergen extracts, i.e., that the drops do not run together. A timer, with an alarm, should be utilized so that all tests, including the histamine and negative control test results are read 15–20 minutes following application. Such timing for test results is recommended even though the histamine control can peak earlier at approximately 8–10 minutes [60].

It is difficult or impossible to develop stable test extracts for certain allergens, in particular, certain foods, e.g., for skin testing to uncooked fruits and vegetables. A prick-to-prick technique is utilized, i.e., first pricking the fresh food with the lancet and then pricking the skin, to test for sensitization to such allergens when clinical allergy is suspected, in particular, oral allergy syndrome. Dry foods, e.g., nuts or cereal, can be pestled in saline and also utilized using the prick-to-prick technique. There can be differences in the degree of skin test reactivity depending on the variety of a fruit or vegetable, how ripe it is, and how it has been stored prior to its use [61].

## Assessing the SPT

Positive and negative controls should be measured first. The negative control excludes the presence of dermographism which, when present, makes the tests difficult to interpret. The histamine control should be positive to make sure that the test materials are applied correctly and to exclude negative SPT results due to potentially interfering medications taken by the test subject (Table 2). The largest diameter of the wheal of each particular test is measured, a positive being a wheal of ≥ 3 mm [62] since the longest diameter is a better estimate of wheal surface area than the mean perpendicular diameters of a skin prick test above a certain value (17 mm^2^). The negative control is no longer used to deduct its size from the positive tests. Including the longest diameter of pseudopods does not increase sensitivity for determining the degree of sensitisation.

Since histamine reactivity in the skin varies among individuals, independent of skin test reactivity to allergens [52], the skin test results to allergens should not be related to the size of the histamine reaction [63]. The size of the wheal is not solely due to histamine as some subjects with positive SPT reaction show no significant histamine release to these allergens as assessed by microdialysis technique [64]. Reproducibility is greater when only the diameter of the wheal, and not the associated erythema, is measured [65,66]. In order to achieve a permanent record, the size of the wheal may be outlined with a pen, blotted onto a cellophane tape, and transcribed onto paper and/or stored electronically.

## Clinical relevance

The SPT confirms sensitization to a specific allergen, however, its clinical relevance must be interpreted based on the medical history and clinical symptoms (see Additional file 1: Table S3). Sometimes, conjunctival, intranasal, oral or even bronchial challenge provocation tests are performed to support clinically relevant sensitivity. The clinical relevance of SPT results varies, depending on the allergen utilized and the population tested. For example, sensitization to house dust mite occurs in some subjects in the absence of clinical relevance [4].

## Extracts

Allergen extracts ideally should be standardized based on the content of the major and minor allergenic determinants since not all patients are allergic to each antigen within an individual extract. They should have batch-to-batch consistency and the skin test results should be comparable when the same extracts from different manufacturers are utilized. Since allergen extracts are biological mixtures containing a variety of different proteins, glycoproteins and polysaccharides, this is difficult to achieve. In fact, SPT results obtained with the same allergen with extracts from different manufacturers vary [67-72]. Thus, when SPT results are compared, the allergen extract utilized should be obtained from the same manufacturer. Likewise, effective allergen immunotherapy requires specified quantities of allergenic components in the extracts used for immunotherapy.

The fact that accurate standardization of extracts is of great importance for their quality has led manufacturers to implement extensive protocols for standardization. Each company uses its own in-house reference material and unique units to express potencies. Such variability among different manufacturers leads to an inability to compare different products and test results. However, since most major allergens of relevant allergens have been identified during the last several decades, the concept introduced is to quantitate the major allergens in each of the individual extracts. Such quantification will allow comparison between products by different manufacturers. In 2001, an EU funded project, the CREATE project, was introduced to encourage standardisation of allergen extracts based on their content of major allergen(s). The project evaluated the use of recombinant allergens as reference materials for major allergen measurements [73]. Another attempt to standardize extracts involved the development of recombinant allergen extracts. Even though some of these recombinant allergens show comparability to allergen extracts derived from source material [74-76], they only cover a limited number of allergens and are still under investigation.

Extracts should not contain preservatives which can cause false positive reactions, e.g., sodium merthiolate. Nor should they be mixed with other allergens, e.g. house dust mite with dog dander extract. When testing with non-commercial allergens, there is a real need to use control tests in non-allergic subjects to compare the results with subjects who are allergic. For certain plant allergens, especially for fresh foods and vegetables, the prick-to-prick method is more reliable than using manufactured extracts [61].

The tight regulation of skin test extracts has made their production and registration problematic and costly for the pharmaceutical industry. This has led to gaps in the registration of specific extracts in certain European countries.

## Pan-European skin prick test panel for respiratory allergens

The authors suggest that a standard SPT panel for inhalant allergens, based on the GA^2^LEN study [3], be utilized throughout Europe. This panel includes the following 18 allergens: hazel (*Corylus avellana*), alder (*Alnus incana*), birch (*Betula alba*), plane (*Platanus vulgaris*), cypress (*Cupressus sempervirens*), grass mix (smooth meadow grass/*Poa pratensis*, cock’s foot grass/*Dactilis glomerata*, perennial rye grass/*Lolium perenne*, timothy grass/*Phleum pratense*, meadow fescue/*Festuca pratensis*, meadow oat grass/*Helictotrichon pretense*), Olive (*Olea europaea*), mugwort (*Artemisia vulgaris*), ragweed (*Ambrosia artemisiifolia*), *Alternaria alternata* (*tenuis*), *Cladosporium herbarum*, *Aspergillus fumigatus*, *Parietaria*, cat, dog, *Dermatophagoides pteronyssinus*, *Dermatophagoides farinae*, and cockroach (*Blatella germanica*). Allergens can be supplemented as necessary for regional or for particular patient needs.

## Interpretation of SPT results

SPT results should be appropriately interpreted based on clinical symptoms, medical history, and, where necessary, other test results (specific IgE antibody measurements) in order to assess possible allergy to a specific allergen. The probability of a given sensitization to be clinically relevant depends on the type of allergen and country where the patient lives [4]. The clinical relevance of any detected sensitization should be determined by an allergologist after taking a complete history and performing a physical examination. When SPT results and the history are inconclusive, provocation tests may help to determine the clinical relevance of the SPT sensitization, e.g., before initiation of a specific immunotherapy.

SPT is highly specific and sensitive, 70-95% and 80-97%, respectively, to diagnose inhalant allergies [76]. The positive predictive value to diagnose allergic rhinitis based only on the clinical history is 77% for persistent allergy and 82-85% for intermittent seasonal allergy [17]. This increases to 97-99% if SPT is utilized [17].

The negative predictive value of a negative SPT and in vitro IgE antibody test for cat allergen are identical at 72-75% for cat allergy [42]. A negative SPT for *Dermatophagoides pteronyssinus* has a negative predictive value in older adults of 90%-95%. However, the positive predictive value ranges from 29% to 43% in older subjects and 77% to 100% for younger subjects [23].

Sensitivity and specificity are lower for food allergens, ranging from 30-90% and 20-60%, depending on the type of allergen and methods utilized, i.e. pricking with extracts vs. prick-to-prick techniques described earlier [77]. Double-blind placebo-controlled challenge studies in children demonstrate that SPT possesses a positive predictive value of 76% and 89% for clinical reactions to cow’s milk and hen’s egg, respectively [78].

The objective value of SPT for drug allergy depends on the tested drug. In most cases, a positive SPT makes drug allergy very probable; whereas a negative result does not necessarily indicate that the patient will not react on challenge to the drug [79]. However, for penicillin, the negative predictive value is high. In 98.5% of patients with a negative SPT, no type I allergy was observed upon challenge while the remaining 1.5% of patients had mild and self-limiting reactions, e.g., urticaria [80]. In many cases, intradermal testing is appropriate after negative SPT. Some drugs, e.g., muscle relaxants or opioids may cause SPT false-positive results. When evaluating patients for IgE-mediated drug allergy to antibiotics other than penicillin, SPT should be performed with the unadulterated pharmaceutical agent. Late readings (> 24h) of SPTs and especially intradermal skin tests are very valuable in the clarification of adverse drug reactions.

For suspected insect venom allergy, intradermal tests are the primary mode for detecting sensitization. SPT is performed prior to intradermal testing.

Sensitizations to aeroallergens, as measured by SPT, may precede symptomatic allergy. Prospective studies show that 30-60% of such subjects become allergic depending on the type of allergen tested and the time to follow-up [81,82]. Furthermore, sensitization can exist to an allergen that is no longer clinically relevant.

## SPT in epidemiologic studies

Sensitization rates vary depending on the geographic region as measured in population-based and in patient-based studies like the European Community Respiratory Health Survey (ECHRS), the International Study of Asthma and Allergies in Childhood (ISAAC), and the GA^2^LEN Pan-European skin prick test study. Exposure rates and genetic differences can explain some of these variations [83,84]. With increased human mobility, differences in exposure to various flora or alterations in the allergenicity of pollen, possibly caused by pollution [85,86], and changes in sensitization occur over time [87]. Longitudinal studies investigating sensitization over time provide data on such trends [88,89].

The same allergen extracts and ideally even the same batches of extracts should be utilized when comparing a test result from one place to another or over time. For epidemiological studies, the standard prick test panel should be utilized to ensure comparability with the GA^2^LEN study results (see Table 1; [3]). Distribution of standard operating procedures for both technical aspects and data collection assures the highest degree of comparability. Studies of allergic sensitization should take place over an extended period of time, ideally, a year, since (i) skin test reactivity increases during the pollen season [90] and (ii) allergic individuals tend to seek care when they have symptoms. This can skew detected prevalence of sensitization in such studies. It is also important to note the seasonal differences of various allergens based on geographic locations which can further distort sensitization rates between countries.

## Future directions to use skin tests for allergy diagnosis

More than 1,800 allergenic molecules are identified (http://www.allergome.org/script/statistic.php; [91]). The use of recombinant allergen molecules for SPT should improve sensitivity and standardization of SPT by reducing non-specific reactivity due to irritant compounds contained in biologically derived extracts, in particular, food extracts [92]. New in vitro techniques using recombinant molecules and micro-technology may permit testing of hundreds of compounds simultaneously, thus improving diagnostic possibilities and potentially even eliminating the need for skin testing [93].

## Needs for further research

Further studies are needed to (1) compare extracts from different manufacturers, (2) investigate intra subject variability, and (3) determine the relevance of SPT for pan-allergens. Since new allergens are being identified in Europe, e.g., acerola (*Malpighia glabra*), and others are becoming more prevalent, e.g., *Lepidoglyphus destructor*, new studies should also investigate the relevance of these allergens.

## Conclusion and outlook

There is general agreement that the core diagnostic test for type I immediate allergy, i.e. the SPT, should be further standardized to include standardized procedures and allergen panels. Additional allergens can be added to this “core”, when indicated. Such standards are likely to: (1) improve the quality of patient diagnosis and care, and (2) reduce variability of results and thus make test results comparable. Further studies are necessary to define worldwide standards for allergen extracts.

## Abbreviations

GA^2^LEN: Global Allergy and Asthma European Network; IgE: Immunoglobuline E; SIT: Specific immunotherapy; SPT: Skin prick test; mm: millimetre; RAST: Radioallergosorbent test.

## Competing interests

No conflicts of interest exist for any of the authors.

## Authors’ contributions

Conceived and designed the European SPT study: LMH, MB, GB, UD, SD, WF, MG, TH, ATB, SW. Wrote the manuscript: LMH, RL, AM, KCB. Critically reviewed and revised the manuscript: MB, GB, UD, SD, WF, MG, TH, ATB, SW, HM. Final language editing: SD. Agreement with manuscript and conclusions: all. Designed the figures and tables: LMH, RL. All authors read and approved the final manuscript.

## Authors’ information

Lucie Heinzerling, Karl-Christian Bergmann, Megon Bresciani, Guido Burbach, Ulf Darsow, Stephen Durham, Wytske Fokkens, Mark Gjomarkaj, Tari Haahtela, Ana Todo Bom, Stefan Wöhrl: GA^2^LEN member.

## Supplementary Material

Additional file 1: Table S3 Skin prick test panel – inhalant allergens. Click here for file
