# Supplementary material for: The skin prick test – European standards
Source: Clin Transl Allergy. 2013 Feb 1;3:3. doi: 10.1186/2045-7022-3-3 (PMC3565910; doi:10.1186/2045-7022-3-3)
Supplement: Additional file 1: Table S3 — Skin prick test panel – inhalant allergens. [file 2045-7022-3-3-S1.doc]

**Table S3**

# Skin prick test panel – inhalant allergens

Pat.-ID

Date:_________________

Clinical symptoms: □ Rhinitis

□ Atopic Dermatitis

□ Asthma

□ Food Reactions

□ Urticaria

Season of symptoms: _________________________________

□ All year

□ Symptoms intermittent independent of season

□ No symptoms of atopic dermatitis, allergic rhinitis/asthma or food allergy

| Allergen extract | **Largest diameter** Ø (mm) of wheal  after 15 min | **Relevance** Not relevant (0)/ Relevant (1)/ Former Relevance (2)/ Unknown (3) * |
| --- | --- | --- |
| 1. Histamine |  |  |
| 2. Negative control |  |  |
| 3. Hazel |  |  |
| 4. Alder |  |  |
| 5. Birch |  |  |
| 6. Plane |  |  |
| 7. Cypress |  |  |
| 8. Grass mix |  |  |
| 9. Olive |  |  |
| 10. *Artemisia* |  |  |
| 11. *Ambrosia* |  |  |
| 12. *Alternaria* |  |  |
| 13. *Cladosporium* |  |  |
| 14. *Aspergillus* |  |  |
| 15. *Parietaria* |  |  |
| 16. Cat |  |  |
| 17. Dog |  |  |
| 18. *Derm. pteron.* |  |  |
| 19. *Derm. farinae* |  |  |
| 20. *Blatella* |  |  |
|  |  |  |
|  |  |  |

*Only fill in when positive results. Not relevant: No related allergic symptoms/ Relevant: Related allergic symptoms/ Former relevance: Previously related allergic symptoms
